# Crosslinked 4-Vinylpyridine Monodisperse Functional Microspheres for Sorption of Ibuprofen and Ketoprofen

**DOI:** 10.3390/polym14102080

**Published:** 2022-05-20

**Authors:** Marta Grochowicz, Łukasz Szajnecki, Magdalena Rogulska

**Affiliations:** Department of Polymer Chemistry, Institute of Chemical Sciences, Faculty of Chemistry, Maria Curie-Sklodowska University, 20-031 Lublin, Poland; l.szajnecki@umcs.pl (Ł.S.); mrogulska@umcs.lublin.pl (M.R.)

**Keywords:** polymeric microspheres, porous polymers, SPE, functional polymers, sorption, ibuprofen, ketoprofen

## Abstract

Nowadays, ibuprofen and ketoprofen are widely used over-the-counter medications to treat inflammation, fever, or pain. Their high consumption and improper disposal cause them to get into the environment and often pollute surface water. In this study, the new polymeric porous microspheres based on 4-vinylpyridine (4VP) are presented as effective sorbents for ibuprofen and ketoprofen preconcentration and removal. The porous microspheres were obtained via seed swelling polymerization with the use of two types of methacrylate crosslinkers, i.e., trimethylolpropane trimethacrylate (TRIM) and 1,4-dimethacryloiloxybenzene (14DMB). Additionally, as a reference sorbent, a copolymer of styrene and divinylbenzene was obtained. Porous structure investigations showed that the microspheres possess a specific surface area of about 100 m^2^/g, but noticeable differences were observed in their internal topography depending on the type of crosslinker used. Moreover, the porous structure of dry and swollen microspheres differs significantly. Swollen copolymers reveal the presence of micropores. The 4VP microspheres are characterized by high thermal stability; their initial decomposition temperature is about 300 °C. The performance of the 4VP copolymers as sorbents in aqueous solutions of drugs was evaluated in static and dynamic modes at three pH values of 3, 7, and 11. The highest sorption efficiency was obtained for ibuprofen and ketoprofen in pH 3. Both 4VP copolymers indicate the high sorption capacity in a static sorption as follows: towards ketoprofen of about 40 mg/g whereas towards ibuprofen of about 90 mg/g and 75 mg/g on copolymer crosslinked with trimethylolpropane trimethacrylate and 1,4-dimethacryloiloxybenzene, respectively. The recovery of ibuprofen and ketoprofen after dynamic sorption experiments was higher than 90%.

## 1. Introduction

Ibuprofen, that is 2-[4-(2-methylpropyl) phenyl] propanoic acid and ketoprofen—2-(3-benzoylphenyl) propanoic acid, belong to the broad group of non-steroidal anti-inflammatory drugs (NSAIDs). Most probably, they work by non-selective inhibition of cyclooxygenase enzymes that promote inflammation [1]. In addition to their anti-inflammatory effect, they also have an analgesic effect. Ibuprofen and ketoprofen are commonly used to treat mild to moderate muscle pain, dysmenorrhea, fever, and inflammation. Moreover, ketoprofen is used to treat rheumatoid arthritis, osteoarthritis, postoperative pain, and postpartum pain. The high consumption of ibuprofen, as well as low-dose ketoprofen, results from their high availability; they are over-the-counter drugs (OTC). In many European countries, pharmaceuticals containing ibuprofen can also be purchased outside pharmacies [2]. According to Industry Research, the global ibuprofen market was valued at USD 89 million in 2020 and is expected to reach USD 109 million by the end of 2027 [3]. High consumption, incorrect storage, and bad disposal of medicinal substances lead to increasing environmental pollution by drugs and their metabolites. NSAIDs are detected in surface waters, groundwater, and even bottled mineral waters [4,5]. Traditional wastewater treatment plants are unable to remove drugs with low levels of ppm or ppb in the wastewater. In many European, African countries, and the US, NSAIDs are still detected in the waters flowing from wastewater treatment plants [6,7,8,9,10]. Since ibuprofen and ketoprofen, as cyclooxygenase inhibitors, interact with the human endocrine system, the amount of their consumption should be carefully controlled. In addition to affecting the human body, these substances and their biologically active metabolites adversely affect aquatic organisms.

In order to know in detail, the effects of pharmaceuticals present in the environment on living organisms, their quantity must first be precisely determined. Conducting a quantitative analysis of medicinal substances present in lakes or rivers in very low concentrations requires a preliminary concentration of the samples. Only preconcentrated samples can be analyzed using chromatographs working with sensitive mass spectrometry detectors, but when the concentration of samples is high enough, it is also possible to use cheaper and more widely available UV detectors.

Solid-phase extraction (SPE) is very often used to preconcentrate and clean up water samples. Among the sorbents mainly used in SPE to concentrate ibuprofen and ketoprofen are commercially available materials based on modified silica, e.g., Strata X, C18, and Oasis hydrophilic-lipophilic balanced (HLB) sorbents [11,12,13,14,15]. However, they cannot be used in all the pH ranges and only single-use is recommended, which is not environmentally friendly. Polymer sorbents are a useful alternative because of their properties such as chemical resistance against organic solvents, acids and bases, mechanical strength, permanent porosity, variety of functionalities, high selectivity, and reusability. Among polymeric sorbents, much attention has been paid to molecularly imprinted polymers (MIP) that should be of enhanced selectivity than their nonimprinted counterparts. MIP towards ibuprofen were synthesized using different functional monomers, e.g., methacrylic acid [16], 2-vinylpyridine [17,18], and towards ketoprofen using 4-vinylpyridine [19], methacrylic acid, and acrylamide [20]. For MIP synthesis, mainly bulk or precipitation polymerization is used. Polymers obtained via the bulk technique are in the form of blocks that have to be grounded before being used in SPE. This process generates a lot of waste, and the particles are of a broad size distribution. On the other hand, particles produced by the precipitation method are in the shape of microspheres, but their diameters are lower than 5 µm, which is unfavorable for SPE sorbents.

In this study, we present new 4-vinylpyridine crosslinked porous microspheres that can be successfully applied as sorbents for ibuprofen and ketoprofen preconcentrating and also removal from water solutions as an alternative to MIP and silica-based sorbents. The 4-vinylpyridine copolymers were obtained by seed swelling polymerization in the form of microspheres with diameters of about 9 µm and a narrow particle size distribution. The aim of this work was to assess the usefulness of new copolymers in static and dynamic sorption experiments with water solutions of ketoprofen and ibuprofen.

## 2. Materials and Methods

### 2.1. Materials

Styrene (St) (99%), 4-vinylpyridine (4VP) (95%), trimethylolpropane trimethacrylate (TRIM) (90%), divinylbenzene (DVB) (85%), 2,2′-azobis(2-methylpropionitrile) (AIBN) (98%), ibuprofen (IBU) (99%), ketoprofen (KETO) (99%), aspirin (ASP) (99%), and sodium lauryl sulfate (SDS) (99%) were obtained from Sigma-Aldrich. Toluene, methanol, ethanol, tetrahydrofuran (THF), acetone, and methanoic acid were from POCh (Gliwice, Poland). The 1,4-dimethacryloiloxybenzene (14DMB) was obtained in our laboratory according to the earlier described procedure [21]. Polystyrene standards for inverse size exclusion chromatography (ISEC) experiments were obtained from Toyo Soda (Tokyo, Japan). THF, acetonitrile, and methanol of HPLC-grade used in chromatography experiments were from Merck Milipore. Monomers: 4VP, TRIM, and styrene were purified of inhibitor by vacuum distillation and stored in a refrigerator until use. Other reagents were used as received without further purification.

### 2.2. Preparation of Poly(4VP-co-14DMB) and Poly(4VP-co-TRIM) Copolymers

**Figure 1:**
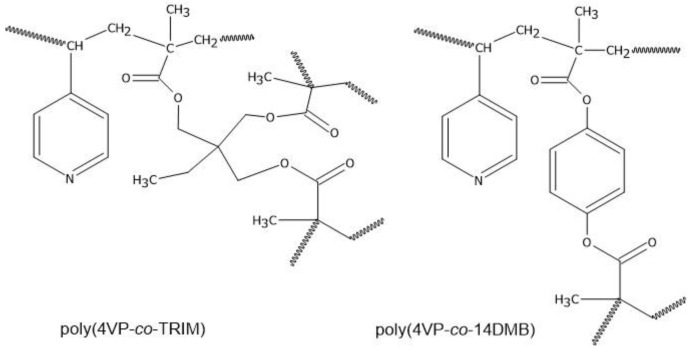
The schematic chemical structures of obtained 4VP copolymers.

The synthesis of two types of microspheres of 4-vinylpyridine (4VP) crosslinked with 1,4-dimethacryloiloxybenzene (14DMB) and trimethylolpropane trimethacrylate (TRIM) was carried out with the use of seed swelling polymerization [22,23]. Molar ratio of 4VP to 14DMB and 4VP to TRIM was 2:1; AIBN was used as a polymerization initiator; toluene acted as an activator of polystyrene seed and pore-forming agent. As the start polymer seed, polystyrene microspheres (PS) were used. Dispersion polymerization was chosen as a suitable method for making PS particles with M_W_ = 19,500 Da. Seed swelling polymerization was carried out in 0.25% water solution of SDS in two steps. Firstly, the swelling of PS seed with monomers, initiator, and toluene mixture took place at 30 °C for 24 h at 150 rpm. In the following step, the temperature was increased to 71 °C and the polymerization was carried out for next 24 h. After the reaction was completed, the obtained spheres were washed with methanol, hot water, and ethanol, respectively, and finally extracted with hot THF for 4 h to remove seed particles. In Figure 1, the chemical structures of copolymers are presented. Additionally, to make the comparison of the chosen properties of 4VP copolymers with the traditional St-DVB sorbent, poly(St-*co*-DVB) microspheres were synthesized according the above-presented method, using molar ratio of St to DVB of 1:1.

### 2.3. Methods of Analysis

ATR-FTIR spectra were recorded using the Tensor 27 spectrometer (Bruker, Germany), equipped with a diamond crystal. The spectrum was made in the spectral range of 600–4000 cm^−1^ with a resolution between 4 cm^−1^ and 16 scans per spectrum.

Carbon-13 Cross-Polarization Magic-Angle Spinning Nuclear Magnetic Resonance (^13^C CP/MAS NMR) measurements were completed on a Avance 300 MSL instrument (Bruker, Germany). The number of scans was 2048, and the spin rate was 7300 Hz.

The morphology and the internal structure of microspheres were examined using scanning electron microscope SEM (FEI Quanta 3D FEG, Fei, Hillsboro, Oregon, USA). Polymeric particles were coated with a thin layer of gold. To determine the number average diameter (D_n_) and coefficient of variation (CV) Morphologi G3 particle analyzer (Malvern, UK) was used.

Parameters characterizing the porosity of the copolymers were determined by nitrogen adsorption-desorption method (LTNA) at −196 °C using ASAP 2420 analyzer (Micromeritics, Norcross, GA, USA). Before measurements, samples were degassed at 60 °C under vacuum. The specific surface area, S_BET_, was evaluated using the standard Brunauer-Emmett-Teller (BET) method for the nitrogen adsorption data in the range of a relative pressure p/p_0_ from 0.05 to 0.25, assuming that the area of a single nitrogen molecule is 16.2 Å^2^. The total pore volume was estimated from single-point adsorption at a relative pressure of 0.985. The pore size distributions (PSD) were obtained from the desorption branch of the isotherm using the Barrett-Joyner-Halenda (BJH) procedure [24].

In a swollen state the copolymers were characterized by inverse size exclusion chromatography (ISEC) [25] using the following as probe molecules: toluene, acetophenone, butyrophenone, dimethyl phthalate, diethyl phthalate, dipropyl phthalate, dibutyl phthalate, dioctyl phthalate, dinonyl phthalate, dilauryl phthalate, and monodisperse polystyrene standards of molecular weights (M_W_) of 580, 2450, 5100, 11,600, 30,300, 68,000, 120,000, 390,000, 750,000, 1,260,000, and 2,750,000 Da. The standards were dissolved in THF. Pure THF was also used as the mobile phase during ISEC measurements. Microspheres under study suspended in methanol were slurry packed into stainless HPLC columns (100 mm × 4.6 mm i.d.) using Pack in a Box system (SSI, Woodlawn, MD, USA). Methanol was used as packing solvent; the filling process was performed under the constant pressure of 20 MPa.

The diameter of the probe molecules (Φ, in Å) was calculated from the following Equation (1) [25]:

(1) $$\Phi =0.62\times M_{W}^{0.59}$$

where $M_{W}^{0.59}$ is the molecular weight of the probe molecule.

The cumulative pore size distribution was determined from the plot *1—K_0_(EC)* versus *log*Φ, where *K_0_(EC)* is the distribution constant in ISEC calculated as follows (2) [26,27,28]:

(2) $$K_{0}\left(EC\right)=\frac{V_{R}-V_{0}}{V_{p}}=\frac{V_{R}-V_{0}}{V_{i}-V_{0}}$$

where: *V_R_*—the retention volume of the probe; *V*_0_—the interstitial volume equal to the retention volume of a totally excluded molecule; *V_i_*—the retention volume of a totally included molecule; *V_p_*—the pore volume.

The simultaneous thermogravimetric analysis (TG) was performed on an STA 449 F1 Jupiter (Netzsch, Selb, Germany) at the heating rate of 10 K/min, in the temperature range of 30–700 °C, with the sample mass of 10 mg in air atmosphere. The gas flow was 20 mL/min. As the reference, the empty Al_2_O_3_ crucible was used.

For both static and dynamic sorption experiments, and for calibration curve, the stock solution of ibuprofen and ketoprofen was prepared in acetonitrile. The concentration of ibuprofen and ketoprofen was of 5.07 mg/mL and 5.06 mg/mL, respectively. The water solutions were prepared by a dilution of stock solution.

The concentration of drugs was calculated on the basis of chromatographic measurements. Experiments were performed using the high-performance liquid chromatograph HPLC (Dionex Ultimate 3000; Dionex, Sunnyvale, CA, USA) equipped with a UV detector. Chromatographic conditions were as follows: Hypersil Gold column (150 × 4.6 I.D.) was thermostated at 30 °C; mobile phase: acetonitrile/0.2% methanoic acid (60/40 *v/v*); flow rate 1 mL/min; the injection volume was 10 µL.

Static sorption experiments of ibuprofen and ketoprofen were performed at room temperature from water solutions. The concentration of drugs solution was 20 mg/L. In total, 20 mL of solution was transferred into the glass vial containing 50 mg of 4VP or poly(St-*co*-DVB) microspheres. The mixture was stirred for 10 min, then centrifuged and 2 mL of supernatant was taken to HPLC analysis to assess the concentration of drugs remained in the solution after sorption. The static experiments were carried out from water solutions with pH of 3, 7, and 11. The sorption capacities were calculated according to Equation (3) [17], as follows:

(3) $$sorption\;capacity\;\left[mg/g\right]=\frac{\left(c_{o}-c_{e}\right)V}{w}$$

where: *c_o_*—the initial drug concentration before the sorption; *c_e_*—the final drug concentration remaining in solution after contact with sorbent; *V*—the volume of the solution; *w*—mass of the sorbent.

Solid-phase extraction (SPE) experiments were made using glass cartridges filled with 50 mg of sorbents. Frits were placed below and above the sorbent bed. SPE process was carried out in a glass vacuum chamber at room temperature. Firstly, the cartridges were conditioned rising 10 mL of methanol, then 30 mL of water. Next the appropriate volume of drug water solutions (of pH of 3, 7, 11) was passed through the sorbent at flow rate of 1 mL/min. The column was vacuum dried and then washed with 5 mL of water. The retained drugs were eluted with 5 mL of methanol and injected into HPLC. After each use, the SPE columns were regenerated by washing with 10 mL of methanol and 10 mL of water, respectively.

## 3. Results

### 3.1. Characterization of Copolymers

Copolymeric poly(4VP-*co*-14DMB) and poly(4VP-*co*-TRIM) microspheres were obtained via seed swelling polymerization. It is a suitable method for preparing polymeric beads with small diameters and narrow particle size distribution, which are important parameters determining the use of such microspheres as sorbents in chromatographic applications. As a functional monomer, 4VP was used, whereas 1,4-dimethacryloyloxybenzene or trimethylolpropane trimethacrylate acted as a crosslinking agent. In Figure 1, the schematic chemical structures of the obtained copolymers are presented. The proposed composition of monomers enables the synthesis of the crosslinked copolymers with the presence of the following polar functional groups: the carbonyl one derived from 14DMB or TRIM monomer and basic pyridine one from 4VP monomer that makes the copolymers less hydrophobic than the traditional poly(St-*co*-DVB) sorbent. On the other hand, the aromatic rings which are able to create π-π interactions are still present in the copolymer’s structure.

**Figure 2:**
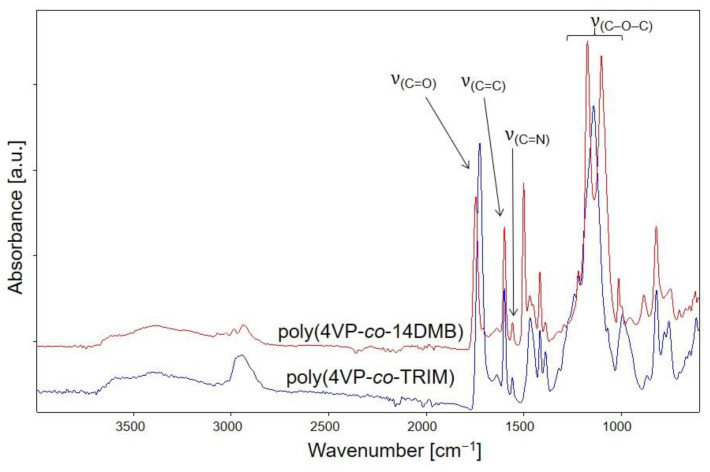
ATR-FTIR spectra of obtained 4VP copolymers.

The chemical structures of poly(4VP-*co*-14DMB) and poly(4VP-*co*-TRIM) sorbents were evaluated by ATR-FTIR measurements. In Figure 2, the obtained spectra of the copolymers are shown. The absorption bands observed between 1599 cm^−1^ and 1558 cm^−1^ derive from C=C and C=N stretching vibrations of the pyridine ring and confirm that 4VP units are present in the copolymer network. On the other hand, a significant absorption band at 1745 cm^−1^ is attributed to the C=O stretching vibration from 14DMB and TRIM units. The second characteristic bands derived from the used crosslinkers are those in the region of 1173–1101 cm^−1^ (COC stretching vibration). Thus, it was confirmed that the functional 4VP monomer and crosslinked 1,4DMB or TRIM monomers took part in the polymerization reaction.

**Figure 3:**
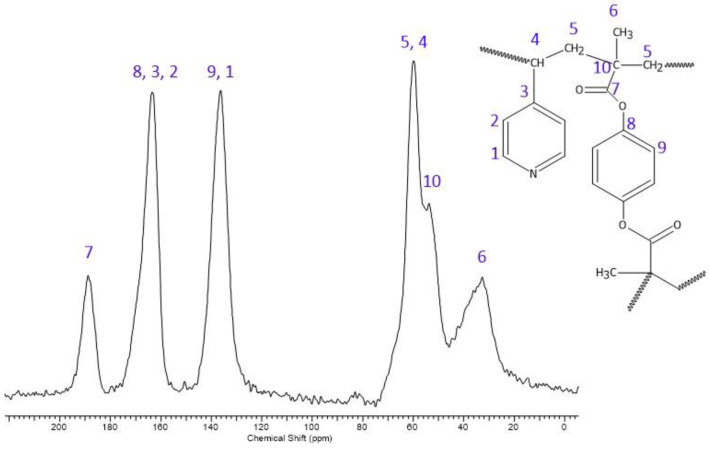
^13^C CP/MAS NMR spectrum of poly(4VP-*co*-14DMB).

The obtained poly(4VP-*co*-14DMB) is a brand-new material, so for its detailed chemical structure characterization, the ^13^C CP/MAS NMR analysis was also performed. It confirmed the proposed in Figure 1 structure of the synthesized adsorbent. Taking into account the spectrum given in Figure 3, the broad signals derived from the resonance of carbon atoms are as follows: δ = 188 ppm (C=O), δ = 163 ppm (Ar C_8,3,2_), δ = 136 ppm (Ar C_9,1_), δ = 59 ppm (-CH_2_-, -CH-), δ = 52 ppm (quaternary C), and δ = 32 ppm (-CH_3_).

Additionally, to assess the amount of 4VP monomer incorporated into copolymeric structures, elemental analysis was performed. As is seen in Table 1, the percent of nitrogen in the structure of poly(4VP-*co*-14DMB) and poly(4VP-*co*-TRIM) is 5.56% and 4.32%, respectively. The used for polymerization, 4VP:crosslinking agent-relative molar ratio was 2:1. The calculation based on the elemental analysis results shows that in the prepared microspheres, the 4VP ratio to 14DMB is 1.72:1, and the 4VP ratio to TRIM is 1.5:1. Both these molar ratios are close to the assumed ones. It also confirms that the obtained copolymeric networks consist of units derived from 4VP and crosslinkers.

**Figure 4:**
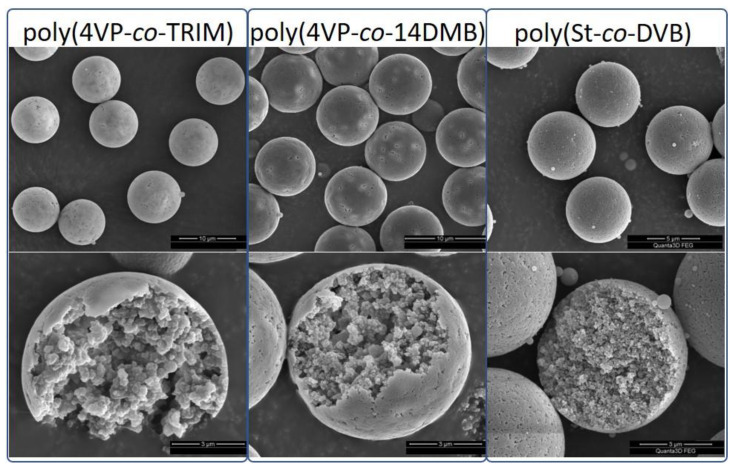
SEM images of 4VP copolymers and poly(St-*co*-DVB).

The morphology of poly(4VP-*co*-14DMB) and poly(4VP-*co*-TRIM) microspheres was determined by SEM measurements. As is visible in Figure 4 presenting the SEM images of the synthesized copolymers, the particles possess an ideal spherical shape. Their number average diameter is 8.18 µm with a CV of 4.22% for poly(4VP-*co*-14DMB) and 9.25 µm with a CV of 9.83% for poly(4VP-*co*-TRIM). A CV value lower than 10% indicates that the microspheres are nearly uniform in size. For TRIM crosslinked microspheres, the D_n_ value is higher than for those synthesized with 14DMB. The chemical structure of TRIM is more expanded than that of 14DMB, and TRIM possesses three methacrylate groups that can react during polymerization with 4VP. This probably explains why upon the following same polymerization conditions: molar ratio of monomers, amount of PS seed, and toluene, the microspheres produced from TRIM are of higher diameter than those obtained with 14DMB. In addition, images of poly(St-*co*-DVB) microspheres are presented in Figure 4. These microspheres were obtained as reference materials with a highly hydrophobic nature.

On the basis of the obtained images of the surface and the internal topography of the microspheres, it can be clearly stated that the newly synthesized materials show a porous character. The outer layer of microspheres is not smooth; large pores are observed. The interior of the particles reveals a fine-grained, tightly packed structure with empty spaces that create pores connected with tunnels. Such an internal structure is characteristic of crosslinked polymers [29]. However, considering the internal structure of 4VP copolymers and poly(St-*co*-DVB) microspheres, some differences are noticeable. In the interior of the poly(St-*co*-DVB) microsphere, the grains are smaller and more homogenously packed, and the voids between them are smaller than in the case of 4VP microspheres. The probable reason for these differences is the affinity of used monomers to the PS seed. St and DVB are hydrophobic monomers chemically similar to the PS. During the formation of porous copolymeric microspheres, the PS seed is swollen with the monomer mixture and, together with toluene, acts as a porogen. As polymerization proceeds, the phase separation between the formed crosslinked network of the copolymer and the surrounding liquid phase containing monomers and porogens occurs [30]. The resulting microgels, called nuclei, combine and agglomerate to form small domains that are tightly packed and form the final microsphere. When PS is swollen with compatible monomers—St and DVB, the microgels are homogeneously distributed in the liquid phase leading to the homogenous internal grain structure of poly(St-*co*-DVB) with small pores. On the other hand, the 4VP and methacrylate crosslinkers are less hydrophobic than PS seed. The resulting nuclei interact more with each other than with PS and form larger agglomerates connected with larger pores.

**Figure 5:**
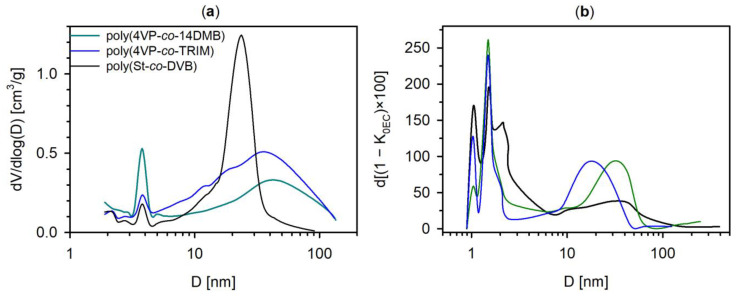
The PSD obtained for studied microspheres from: (**a**) the BJH method using desorption plot; (**b**) the ISEC measurements.

The conducted analysis of porosity by means of N_2_ adsorption-desorption measurements showed that the prepared copolymers possess a developed, permanent porous structure. From Table 1, it follows that 4VP copolymers have a smaller specific surface area and pore volume than poly(St-*co*-DVB). The PSD_max_ for poly(St-*co*-DVB) is also smaller than for methacrylate copolymers (Figure 5a). This finding can be explained by the above-mentioned mechanism of porous structure formation. Since St and DVB monomers are absorbed easier and more homogenously by PS seed than 4VP and methacrylate crosslinkers, the smaller aggregates connected with smaller pores are formed. Considering the differences in S_BET_ of 4VP copolymers, the higher value for poly(4VP-*co*-TRIM) may result from the functionality of a crosslinker. The 14DMB is a bifunctional monomer, whereas TRIM is trifunctional. Using a crosslinker with a higher number of polymerizable methacrylate groups, the phase separation between the microgel and liquid phase is more efficient. The formed microgels are highly crosslinked, and they are not able to dissolve each other as well as in a porogen. Hence, they aggregate into small gel particles which are tightly packed into the final microsphere. When 14DMB is used as a crosslinker, the created microgels are less crosslinked than those with TRIM, the phase separation is hindered, and bigger agglomerates with bigger pores between them are formed, as is visible in Figure 4.

Taking into consideration the PSD presented in Figure 5a, it is visible that they are of bimodal character. The 4VP copolymers are characterized by a broad PSD, whereas the PSD of poly(St-*co*-DVB) is narrower, which is convergent with the SEM images. The 4VP copolymers reveal mesopores and macropores in their internal structure; poly(St-*co*-DVB) is mesoporous. All the studied materials possess the first maximum PSD at about 4 nm. It is a characteristic maximum reported earlier for crosslinked porous polymers synthesized via heterogeneous polymerization techniques [31]. This observation was attributed by Flodin [32] to a very regular structure of the polymer on the micro-level scale and probably corresponds to the pores between nuclei.

Since the studied copolymers were synthesized to be used as sorbents for SPE columns, it is worth knowing their internal porous structure in the swollen state. For this purpose, the copolymeric beads were packed into chromatographic columns and ISEC experiments were performed. The ISEC method allows the determination of the pore size distribution of a sorbent in the range from micropores to macropores with the use of phthalates, alkylphenones, and polystyrene standards. It can be seen from Figure 5b that in the internal structure of all the studied sorbents, micropores are present. The maxima on the PSD-ISEC curves in the range of 1–2 nm are designated as micropores, whereas the maxima at 20–40 nm correspond to mesopores. The pore size distribution determined on the basis of the ISEC measurements is of a different character than the PSD curves obtained from LTNA. It is a typical observation for porous polymers [33,34,35]. Moreover, taking into account the data presented in Table 1, it should be noted that there are significant differences in pore volume for dry and swollen copolymers. Generally, the V_p_ values determined by ISEC are higher than those obtained from LTNA, but the difference is greater for 4VP copolymers than for poly(St-*co*-DVB). In addition, all studied copolymers in swollen states reveal the presence of micropores in their structure. The volume of the micropores (V_micro_) does not exceed 20% of the total pore volume of the 4VP copolymers, but for poly(St-*co*-DVB), the micropore contribution is 35%. Two reasons for the difference in porosity of dry and swollen materials can be considered. Firstly, the micropores present in the structure of a dry copolymer are inaccessible to nitrogen molecules [35]; moreover, it should be emphasized that the values of specific surface area and pore volume were calculated using the 16.2 Å^2^A^2^ surface of a nitrogen molecule, which is normally used for hydrophilic surfaces, whereas the studied copolymers are not a typical hydrophilic material [36]. Secondly, the internal structure of swollen copolymers is changed by the interactions of the polymeric network with THF.

**Figure 6:**
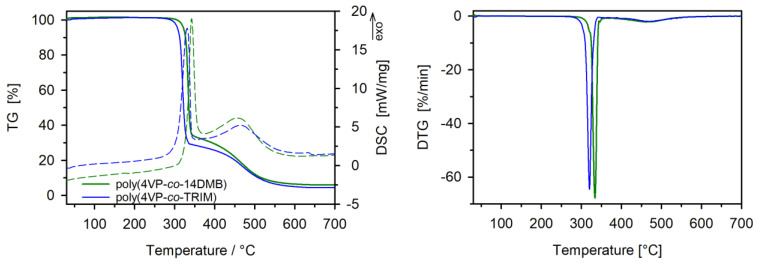
TG and DTG curves and DSC thermograms for 4VP copolymers.

In order to determine the thermal stability of 4VP copolymers in the air atmosphere the TG analysis was carried out. Figure 6 presents the TG, DTG, and DSC curves. The initial decomposition temperature, defined as the 2% mass loss of the sample (*T_2%_*), is 301 °C and 316 °C for TRIM and 14DMB crosslinked copolymer, respectively (Table 2). The thermal decomposition of both copolymers runs in two well-separated stages, which are seen on DTG curves. The first stage is observed in the temperature range of 301 °C–360 °C (with the maximum at *T_max_*_1_) with about 66% and 71% mass loss (Δ *m*_1_*)* for 14DMB and TRIM crosslinked copolymer, respectively. The second one spreads from 361 °C with a maximum of about 460 °C (*T_max_*_2_) and mass loss of about 30% (Δ *m*_2_*)*. From DSC curves, it is visible that the decomposition processes are exothermic. Based on the courses of TG and DTG curves and also the values of *T*_2%_ and *T*_50%_ (the temperature of 50% mass loss), it is seen that the 4VP copolymer crosslinked with 14DMB shows higher thermal stability than that crosslinked with TRIM. Previous detailed studies on the thermal decomposition of poly(4VP-*co*-TRIM) microspheres revealed that their decomposition starts with a depolymerization reaction, during which pyridine is evolved [37]. It is evidenced that pyridine is the catalyst for the hydrolysis of ester bonds. In the case of TRIM crosslinked copolymer, the number of ester bonds is higher than in 14DMB copolymer. Therefore, a higher first mass loss is observed for poly(4VP-*co*-TRIM). The residual mass (*R_m_*) for both copolymers is lower than 5%. Finally, it can be stated that both studied 4VP copolymers possess high thermal stability, and they can be used as sorbents operating in the room as well as at elevated temperatures.

### 3.2. Sorption Studies

**Figure 7:**
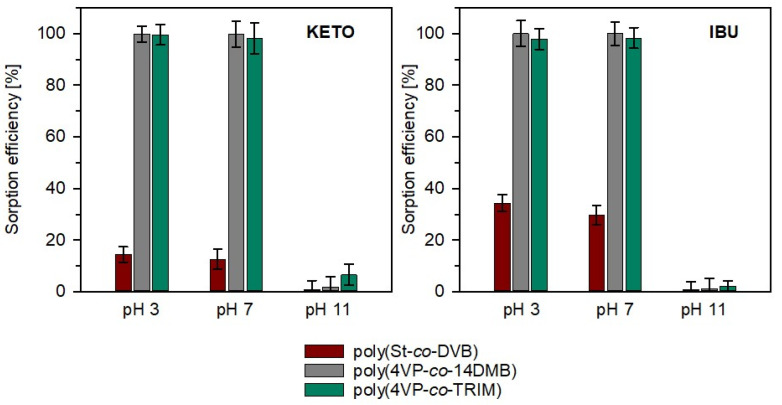
Sorption efficiency of ketoprofen (KETO) and ibuprofen (IBU) on studied copolymers in pH 3, 7, 11; (mean (bar) ± standard deviation (whisker) (*n* = 3)).

The sorption studies of ibuprofen and ketoprofen on 4VP copolymers and poly(St-*co*-DVB) were performed in the following two modes: static and dynamic. Firstly, static experiments were made, where 50 mg of each sorbent was mixed with 20 mL of drug solution. The concentration of IBU and KETO in the water solution was 20 mg/L. It is the maximum concentration that can be operated since the solubility of IBU in water is 21.0 mg/L [38], and KETO is 21.3 mg/L [39]. The effect of solution pH was investigated to achieve the maximum sorption of target drugs. The efficiency of sorption was expressed as the percentage of drug amount adsorbed on the sorbent. As can be seen from Figure 7, the drug sorption is strongly pH-dependent. For both studied drugs, the increase in pH value causes a drop in drug sorption. In pH 11, more than 97% of drugs remain in the water solution, regardless of the sorbent used. On the other hand, in pH 3, 100% sorption of ibuprofen and ketoprofen was obtained on poly(4VP-*co*-14DMB) and almost 100% on poly(4VP-*co*-TRIM), while a significant amount of both drugs remained in the solution contacted with poly(St-*co*-DVB). These results indicate that the sorption of ibuprofen and ketoprofen on 4VP sorbents is the most effective in an acidic environment with pH 3. Ibuprofen and ketoprofen are carboxylic acids; their p*K*_a_ values are 5.30 [38] and 4.45 [39], respectively, so at pH 3, these compounds are protonated. While the sample pH is increased to neutral and then to basic, the carboxylic group is deprotonated, and drug molecules are not able to interact with the sorbent by means of hydrogen bonding. Additionally, the target drugs are aromatic compounds; thus, the π-π interactions may also be responsible for their sorption on all the discussed copolymers. However, when the chemical nature of copolymers will be considered together with the efficiency of sorption, it can be stated that the main role in the sorption mechanism plays hydrogen bonding. Hydrophobic sorbent poly(St-*co*-DVB) may interact with target molecules mainly through π-π interactions. Even in pH 3, the sorption efficiency on this copolymer was much lower than on 4VP copolymers. In their crosslinked networks, besides aromatic rings, the nitrogen atom in the pyridine ring and carbonyl groups are present and can interact by hydrogen bonding. It was also earlier reported that the extraction of acidic NSAIDs from the copolymers containing 2-vinylpyridine is based on hydrogen bonding [17,18].

The sorption results obtained in pH 3 indicated that the sorption capacity of 4VP copolymers in an acidic pH was not exceeded. Therefore, the next experiment was made to assess the 4VP copolymer’s sorption capacity towards ibuprofen and ketoprofen in pH 3. In this case, 24 mg of sorbent was mixed with 50 mL of drug solution with a concentration of 20 mg/L. The calculated sorption capacity of poly(4VP-*co*-TRIM) for ketoprofen is 39.50 mg/g and for ibuprofen 90.81 mg/g, whereas the sorption capacity of poly(4VP-*co*-14DMB) for ketoprofen is 40.13 mg/g and for ibuprofen 75.36 mg/g. These values seem to be very high, and, therefore, 4VP copolymers can be considered the efficient sorbents for ibuprofen and ketoprofen removal from water samples, even with neutral pH.

**Figure 8:**
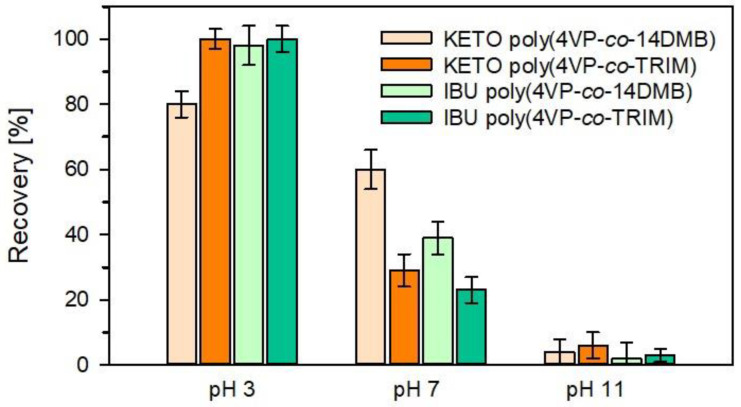
Recoveries obtained for ketoprofen and ibuprofen on 4VP copolymers from solutions of pH = 3, 7, 11; (mean (bar) ± standard deviation (whisker) (*n* = 3)).

Sorption properties of 4VP microspheres in dynamic conditions were tested towards ibuprofen and ketoprofen using the SPE technique. The effect of pH on the recovery of target drugs was also tested in dynamic conditions. In each experiment, 250 mL of a water solution of ibuprofen and ketoprofen with a concentration of 1.1 µg/mL was used. As it is seen from Figure 8, the impact of sample pH has the same trend as in the case of static sorption. The maximum recoveries and the highest preconcentrations were obtained for IBU and KETO in acidic pH on both 4VP sorbents. However, on poly(4VP-*co*-TRIM), the recovery of KETO and IBU was almost 100%, whereas the recovery of KETO on poly(4VP-*co*-14DMB) sorbent was slightly lower and reached about 81%.

**Figure 9:**
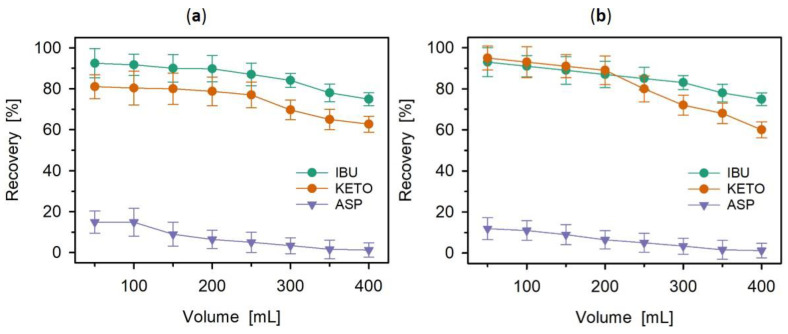
Effect of the sample volume on the recoveries obtained for ibuprofen (IBU), ketoprofen (KETO) and aspirin (ASP) on the (**a**) poly(4VP-*co*-14DMB) and (**b**) poly(4VP-*co*-TRIM) copolymers; (mean (bar) ± standard deviation (whisker) (*n* = 3)).

Moreover, the effect of the sample volume was investigated. In the analysis of environmental samples, large sample volumes are often required to achieve high preconcentration factors and obtain better sensitivity. The experiment was performed at pH 3, as it was verified as the most appropriate for the sorption of tested drugs. The results presented in Figure 9 show that the recovery for ibuprofen and ketoprofen is almost unchanged when the sample volume was from 50 mL to 200 mL and then slightly decreased with the increasing sample volume. The maximum recoveries obtained for ibuprofen and ketoprofen were 92% and 81%, respectively. However, even at a sample volume of 400 mL, the recoveries were still at an acceptable level of about 70%. The observed drop in the recovery along with the increasing sample volume is most probably connected with the exceeding drug sorption capacity. At this point, it has to be emphasized that the effect of sample volume on the recovery was investigated by reusing the same SPE column after its regeneration with 10 mL of methanol after each sorption-desorption experiment. Based on these results, conclusions concerning the stability and reproducibility of sorbents, which are key factors in evaluating their adsorption performance, can be made [40]. As can be seen in Figure 9, the recoveries were stable when the concentration of KETO and IBU in dosed solutions was lower than the sorbent capacity. Both poly(4VP-*co*-14DMB) and poly(4VP-*co*-14DMB) sorbents reused four times (to a volume of 200 mL, Figure 9) do not show a reduction in the extraction efficiencies. Additionally, to estimate the sorbent selectivity to selected drugs from the NSAIDs group that are acidic compounds, aspirin with the mixture of ibuprofen and ketoprofen was also tested. From Figure 9, it follows that both copolymers indicate the highest selectivity towards ibuprofen, slightly lower towards ketoprofen, and the lowest towards aspirin. All these drugs are acidic compounds with aromatic rings in their structures. However, a significant difference in the chemical structures of these three compounds should be considered. The carboxyl group in the aspirin molecule is directly connected to the aromatic ring, thanks to which the π electrons and free-electron pairs from the carboxyl group can participate in resonance with the delocalized electrons of the aromatic ring. In the case of ketoprofen and ibuprofen molecules, such resonance is not possible.

**Figure 10:**
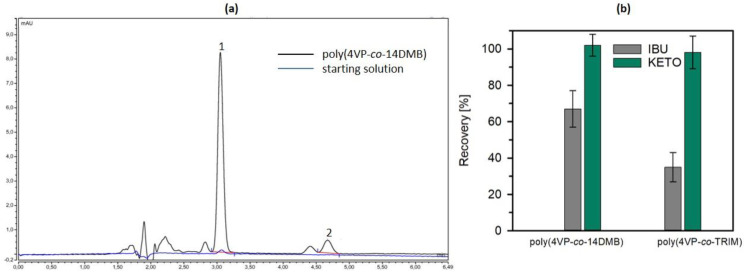
Chromatograms recorded for initial and preconcentrated on poly(4VP-*co*-14DMB) solutions of ketoprofen (1) and ibuprofen (2) (**a**). Recoveries of target compounds obtained after extraction with 4VP sorbents (**b**); (mean (bar) ± standard deviation (whisker) (*n* = 3)).

In order to estimate the possibility of using 4VP sorbents as SPE beds for preconcentration of ibuprofen and ketoprofen from acidic water samples, the adsorption–desorption was made from solutions of low drug concentration, as target compounds have been detected in the water at low µg/L levels [18,19]. In this section, the initial solution concentration of ketoprofen and ibuprofen was between 50 µg/L and 190 µg/L, respectively. In total, 250 mL of solution was dosed to SPE columns filled with 50 mg of sorbents. From the chromatograms presented in Figure 10, it is visible that the peak of ketoprofen is of a very small intensity (below the limit of quantification) and the peak of ibuprofen is invisible when the initial solution was measured. After the preconcentration procedure on poly(4VP-*co*-14DMB), the peaks were more intense. The recovery after SPE is on a very high level for ketoprofen on both sorbents. On the other hand, the extraction of ibuprofen from a very diluted solution was not as efficient as in the case of higher concentration (1.1 mg/L, Figure 8), and a higher recovery was obtained with the use of poly(4VP-*co*-14DMB) sorbent due to the higher preconcentration factor.

## 4. Conclusions

The presented results show the method of synthesis of permanently porous functional microspheres that can be applied as sorbents for effective removal and preconcentration of ketoprofen and ibuprofen from water solutions. The microspheres of 4VP crosslinked with methacrylate aromatic or aliphatic monomer were obtained via seed swelling polymerization. They possess a developed porous structure with a specific surface area of about 100 m^2^/g. It was proved that the porous structure of swollen and dry copolymers differs. In a swollen state, the existence of micropores was revealed. The conducted thermal analysis indicated that prepared microspheres are thermally stable under an oxidative atmosphere up to 300 °C. This makes it possible to use them as sorbents even in experiments carried out at elevated temperatures. The application of poly(4VP-*co*-14DMB) and poly(4VP-*co*-TRIM) microspheres as sorbents for the removal of ibuprofen and ketoprofen from water solutions was tested in static and dynamic modes. In both cases, the sorption efficiency was best when the pH of the used solution was acidic, well below the p*K*_a_ of tested drugs. Since the sorption capacity towards ketoprofen and ibuprofen is high, the proposed 4VP-based sorbents can be used as efficient sorbents for the removal of these drugs from wastewater samples; however, to assess their performance, additional experiments with real samples have to be performed. On the other hand, the discussed sorbents are not suitable for aspirin sorption.

## Figures and Tables

**Table 1:** Percentage of nitrogen content and parameters characterizing porous structure of 4VP copolymers and poly(St-*co*-DVB) determined by means of nitrogen adsorption-desorption method and ISEC.

Copolymer	%N	N_2_ Adsorption-Desorption			ISEC		
		S_BET_ [m^2^/g]	V_p_ [cm^3^/g]	PSD_max_ [nm]	V_p_ [cm^3^/g]	V_micro_ [cm^3^/g]	PSD_max_ [nm]
poly(4VP-*co*-14DMB)	5.56	89	0.26	4/50	0.81	0.12	1-2/17
poly(4VP-*co*-TRIM)	4.32	147	0.29	4/34	0.77	0.14	1-2/30
poly(St-*co*-DVB)	-	225	0.38	4/24	0.51	0.18	1-2/41

**Table 2:** Parameters characterizing the thermal stability of 4VP copolymers.

Sample	*T*_2_*_%_* [°C]	*T*_50_*_%_* [°C]	*T_max_*_1_ [°C]	Δ *m*_1_ [%]	*T_max_*_2_ [°C]	Δ *m*_2_ [%]	*R_m_* [%]
poly(4VP-*co*-14DMB)	316	335	334	66.3	460	28.8	4.9
poly(4VP-*co*-TRIM)	301	322	320	70.7	469	25.8	3.5

## Data Availability

The data presented in this study are available on request from the corresponding author.
